# Predictors of long-term follow-up clinic attendance and continued engagement among survivors of cancer in childhood and adolescence

**DOI:** 10.1007/s11764-025-01903-4

**Published:** 2025-10-15

**Authors:** Ji Yun Tark, Kayla L. Foster, Shawki L. Qasim, Omar Shakeel, Alicia Howell, Mary C. Shapiro, Abiodun Oluyomi, ZoAnn E. Dreyer, Ashley M. Butler, Austin L. Brown, Maria M. Gramatges

**Affiliations:** 1 Baylor College of Medicine, 6620 Main St, Ste 1530, Houston, TX USA; 2 Texas Children’s Hospital, Houston, USA

**Keywords:** Cancer survivorship, Long-term survivor clinic, Adherence, Childhood cancer

## Abstract

**Purpose:**

Survivorship care is vital to screen for and manage late effects, yet follow-up remains suboptimal. We examined factors associated with long-term survivor clinic (LTSC) nonattendance, delayed initiation, and disengagement at a large, diverse pediatric cancer center.

**Methods:**

We analyzed outcomes in 1138 survivorship-eligible childhood cancer survivors diagnosed between 2011 and 2019. Outcomes included (1) LTSC nonattendance (no visit ≥ 2 years post-treatment), (2) delayed LTSC visit initiation (first visit > 5 years from end of treatment), and (3) LTSC visit disengagement (no follow-up after initial LTSC visit). Adjusted odds ratios (aORs) and 95% confidence intervals (CIs) for factors associated with each outcome were estimated by multivariable logistic regression.

**Results:**

The mean age at the end of treatment was 9.3 years (standard deviation ± 5.6); 56% were male, 50% Latino, and 42% had leukemia. Twenty-one percent never attended LTSC. Nonattendance was associated with older age (aOR = 1.15; 95% CI, 1.11–1.18), public (aOR = 2.37; 95% CI, 1.65–3.44), or no insurance (aOR = 2.58; 95% CI, 1.20–5.42). Delayed initiation was linked to higher odds of disengagement (aOR = 1.28; 95% CI, 0.85–1.92). Disengagement was higher among older (aOR = 1.09; 95% CI, 1.05–1.12) and publicly insured (aOR = 2.29; 95% CI, 1.55–3.42) survivors.

**Conclusions:**

Timely and accessible survivorship care is essential to mitigating late effects of cancer treatment. Here, one in five survivors did not receive survivorship care and patients that initiated LTSC early were more likely to remain engaged.

**Implications for Cancer Survivors**

Tailored interventions that support older and under-insured survivors and early referral to LTSC may improve survivor initiation and long-term engagement with survivorship care.

**Supplementary Information:**

The online version contains supplementary material available at https://doi.org/10.1007/s11764-025-01903-4.

## Introduction

Seventy percent of adult childhood cancer survivors develop one or more severe, disabling, or life-threatening chronic health conditions, or ‘late effects’[1]. Excess morbidity and early mortality related to these late effects[2] highlight the critical importance of consistent, lifelong, survivorship-focused care. Survivor-focused care should incorporate individualized risk counseling and health screening for early detection and is a critical need for the growing population of cancer survivors[3]. However, survivor engagement with and access to survivorship care remains low. For example, a study conducted in 8522 Childhood Cancer Survivor Study participants indicated that up to 68.5% of survivors do not receive any survivor-focused care and only 17.8% receive individualized risk assessment and screening[4]. Prior publications have noted older age, race (non-White), lack of insurance, treatment with surgery or radiation alone, non-leukemia diagnosis, absence of stem cell transplantation, and greater distance from the clinic as associated with long-term survivor clinic non-attendance[5–9]. However, the characteristics of survivors who only attend once and never return (disengage) or who delay initiating survivorship care remain understudied. Identifying and addressing barriers to timely and continued survivorship services is a longstanding challenge to optimizing delivery of survivorship care in the United States.

To address the unique health needs of childhood cancer survivors, the Children’s Oncology Group (COG) developed an evidence-based approach to education, screening, and surveillance for late effects in survivors of childhood, adolescent, and young adult cancers[10]. These guidelines serve as the gold standard for delivering survivorship services to individuals diagnosed with cancer in childhood, adolescence, or young adulthood for the vast majority of hospitals and clinics affiliated with the Children’s Oncology Group, including the Texas Children’s Hospital Cancer and Hematology Center (TXCH) Long-Term Survivor Clinic (LTSC). Here, we leverage data from a highly diverse patient population in a large, tertiary children’s hospital facility to understand LTSC referral patterns and factors related to LTSC nonattendance, delayed LTSC visit initiation, and LTSC visit disengagement with survivorship care, with the goal of identifying opportunities for improving access to survivorship care and long-term follow-up guideline adherence[11, 12].

## Methods

### Study setting

The TXCH is part of Texas Children’s Hospital in Houston, Texas and the Pediatric Program of the Dan L. Duncan Comprehensive Cancer Center. A lifelong LTSC was established in 1988 to address the unique health needs of childhood cancer survivors. The TXCH LTSC offers annual risk assessment and screening with the support of Passport for Care, a clinical decision support tool for the COG Long-Term Follow-up Guidelines that is freely available to clinics who care for survivors of childhood cancer[13]. Eligibility for the TXCH LTSC begins at 2 years from end of treatment and includes individuals diagnosed with cancer at ≤ 21 years old and who received treatment either at TXCH or outside TXCH. The clinic serves a diverse population and a wide catchment area that extends beyond the Houston metropolitan area, with an average of ~ 300 new and ~ 700 returning LTSC visits each year.

### Study population

For this study, we included survivors of childhood cancer diagnosed between 2011 and 2019 who received all or part of their cancer treatment at TXCH. Patients were identified by cancer diagnostic codes using the EPIC® SlicerDicer analytics tool and filtered to include only patients with a cancer diagnosis noted as a ‘hospital problem’ and Texas Cancer Registry participation. Cases were verified against the Texas Children’s Cancer Center Patient Registration System and any missing cases were added. Inclusion criteria, in addition to the above, were (1) treated with radiation therapy or chemotherapy, (2) alive and in remission > 2 years after the end of treatment for their childhood malignancy, and (3) residence within a 300-mile radius of TXCH as a proxy for reasonable expectation that the survivors attend clinic in-person (Supplementary Fig. 1). Children who have relapsed or with ongoing cancer treatment needs are followed by their primary oncology team. Children who develop second cancers in survivorship are transitioned back to the primary disease-based team for cancer treatment and return to the LTSC once the treatment is completed. Such children were excluded from the current analysis. In line with our objective to examine adherence to long-term follow-up care, we excluded patients who transferred care to another hospital prior to 2 years from end of treatment, were treated with observation, international referrals, and those treated with allogeneic hematopoietic stem cell transplantation, as such patients are followed in a transplant-specific, separate LTSC. Patients treated with surgery alone were also excluded as there are no specific guidelines available to assess risk based on this exposure. The study was approved by the Baylor College of Medicine Institutional Review Board and was conducted under a waiver of consent.

### Dependent variables

The primary outcome was ‘LTSC nonattendance’ (vs. LTSC attendance at any time-point) among patients at least 2 years from end of treatment, with an ending follow-up date of December 31, 2024 (mean follow-up, 8 years). For those who did attend an LTSC visit, secondary outcomes included (1) delayed LTSC visit initiation, defined as attending the first LTSC visit more than 5 years post-end-of-treatment, and (2) LTSC visit disengagement, defined as attending an initial LTSC visit post-end-of-treatment without subsequent LTSC follow-up (mean follow-up, 4 years from initial LTSC visit). For secondary analyses, survivors who attended their first LTSC visit but then relapsed, died, or moved away were further excluded.

### Data collection

Data extracted from EPIC® included patient demographics (including age, sex, self-reported race/ethnicity), current and historical payer information, current and historical address information, preferred language, and diagnosis. Payer data were classified as commercial, public, or uninsured/self-pay. Public insurance includes Medicaid and CHIP, and only primary insurance was considered. For this analysis, we used the insurance status listed in the electronic health record (EHR) at 2–6 years from end of treatment (the anticipated time of referral to LTSC), using the most recent payer category during this time frame as the predictor variable. Individuals with no available data in this time frame (*n* = 4) or an international payer status (*n* = 1) were further excluded. For data not available through EPIC®, such as diagnosis and treatment data, including verification of primary diagnosis, treatment modality (chemotherapy and radiation, chemotherapy only, or radiation therapy only), and end of treatment date (defined as the last date of oral or intravenous chemotherapy or last day of radiation), were obtained by manual chart review. Primary diagnosis was classified as leukemia, solid tumor, lymphoma, or central nervous system (CNS) tumors. The date of the initial LTSC visit and the most recent LTSC visit were also obtained by manual chart review. The duration of time from the end of therapy to the initial LTSC visit was calculated.

### Area-based determinants of health

The area deprivation index (ADI) is a composite measure of neighborhood socioeconomic disadvantage calculated from 17 U.S. Census variables from the following categories: poverty, employment, housing, and education[14]. Higher ADI scores represent greater socioeconomic disadvantage, with the highest quartile (Q4) indicating the most disadvantaged neighborhoods (≥ 75th percentile of the Texas state distribution). To calculate ADI scores, ArcGIS Pro 2.2 (Esri, Redlands, CA) was used to integrate geographic/geographically-referenced data for 2010 census tracts in the state of Texas, according to published methods[14–16]. The other area-based factors were also total travel time to the LTSC (minutes) and total distance to the LTSC (miles). We used the network analysis tool in ArcGIS Pro 2.2 (Esri, Redlands, CA) to calculate these factors. The patient’s address at the end of treatment represented the origin location while the Texas Children’s Hospital address was the destination location. For patients missing their end of treatment address, we used any of these addresses as the origin location, in order of preference: year before, year after, 2 years before, or 2 years after the end of treatment.

### Statistical analysis

Descriptive statistics were calculated to characterize the study population. Continuous variables were summarized as means and standard deviations (SD), while categorical variables were presented as frequencies and percentages. Missing data (language 0.8%, treatment modality 1.8%, diagnosis 0.4%, total travel time and distance 4.4%, and ADI score 5.4%) were imputed using MissForest[17], a robust method leveraging random forests for imputation[18]. Unadjusted logistic regression was performed to examine crude associations between each covariate of interest and the outcomes. Covariate selection for the multivariable analysis was performed using backward stepwise selection to identify the optimal predictive model for LTSC non-attendance and the secondary outcomes. A multivariable logistic regression model was then constructed using the selected covariates. Results were expressed as adjusted odds ratios (aOR) with corresponding 95% confidence intervals (CI). We assessed multicollinearity among predictors selected via backward stepwise selection and retained in each multivariable model by calculating the generalized variance inflation factor (GVIF)[19]. No evidence of multicollinearity was observed (GVIF < 2). Because the influence of sociodemographic factors on LTSC attendance might vary depending on the other contextual factors, we evaluated potential interactions between age at the end of treatment and race/ethnicity, age at the end of treatment and payer status during the 2–6 years post-treatment, and race/ethnicity and payer status during the 2–6 years post-treatment by including multiplicative interaction terms in separate multivariable models for each outcome. All statistical analyses were conducted in R (version 4.3.2; R Core Team, Vienna, Austria), with statistical significance defined as a two-sided p-value of < 0.05.

## Results

We identified 1143 cases that met the inclusion criteria and were determined to be LTSC-eligible. After exclusion of five cases for unavailable payer data, 1138 remained with a mean age ± standard deviation (SD) of 9.3 ± 5.6 years at the end of treatment (Table 1). Fifty-six percent were male, and the majority was Latino (50%). About half of the survivors had public insurance and 4% were uninsured/self-pay. Distribution of diagnoses and treatment approaches is also shown in Table 1. The mean total travel time ± SD to LTSC visit was 65.2 ± 72.9 min, while the mean total travel distance ± SD was 42.6 ± 67.5 miles. The mean ADI score ± SD was 100.3 ± 18.6 (range −56.3 to 123.5), with mean ADI scores across the first to fourth quartiles as follows: 82.6 ± 21.0, 104.6 ± 4.0, 111.2 ± 2.5, and 116.1 ± 2.4, respectively.

**Table 1:** Univariable and multivariable analyses of factors associated with long-term survivor clinic (LTSC) nonattendance following cancer treatment (*n* = 1138)

Variable	LTSC Attendance^a^ (*n* = 897)	LTSC Nonattendance^b^ (*n* = 241)	Crude OR (95% CI)	*P*–value	Adjusted OR (95% CI)	*P*–value
Age at end of treatment (years), mean (SD)	8.6 (5.3)	11.7 (6.1)	1.10 (1.08–1.13)	< 0.001	1.15 (1.11–1.18)	< 0.001
Sex				0.80		0.58
Male	509 (56.7%)	134 (55.6%)	─		─	
Female	388 (43.3%)	107 (44.4%)	1.05 (0.79–1.39)		1.09 (0.80–1.50)	
Race/ethnicity				< 0.001		< 0.001
Non-Latino White	293 (32.7%)	75 (31.1%)	─		─	
Asian	44 (4.9%)	10 (4.1%)	0.89 (0.41–1.78)		0.95 (0.41–2.05)	
Latino	469 (52.3%)	105 (43.6%)	0.87 (0.63–1.22)		0.56 (0.37–0.84)	
Non-Latino Black	84 (9.4%)	47 (19.5%)	2.19 (1.41–3.38)		1.64 (1.00–2.68)	
Other	7 (0.8%)	4 (1.7%)	2.23 (0.57–7.59)		1.41 (0.33–5.19)	
Payer status 2–6 years from EOT				0.001		< 0.001
Commercial	433 (48.3%)	88 (36.5%)	─		─	
Public	435 (48.5%)	137 (56.8%)	1.55 (1.15–2.09)		2.37 (1.65–3.44)	
Uninsured	29 (3.2%)	16 (6.6%)	2.71 (1.39–5.15)		2.58 (1.20–5.42)	
Treatment modality				< 0.001		< 0.001
Chemotherapy only	654 (72.9%)	158 (65.6%)	─		─	
Chemotherapy + RT	214 (23.9%)	61 (25.3%)	1.18 (0.84–1.64)		0.60 (0.40–0.90)	
RT only	29 (3.2%)	22 (9.1%)	3.14 (1.74–5.60)		2.15 (0.93–5.06)	
Diagnosis				< 0.001		< 0.001
Leukemia	423 (47.2%)	53 (22.0%)	─		─	
CNS tumor	100 (11.1%)	33 (13.7%)	2.63 (1.61–4.27)		2.73 (1.33–5.43)	
Lymphoma	134 (14.9%)	55 (22.8%)	3.28 (2.14–5.02)		2.49 (1.57–3.95)	
Solid tumors	240 (26.8%)	100 (41.5%)	3.33 (2.31–4.83)		5.23 (3.44–8.06)	
Language				0.14		
English	723 (80.6%)	204 (84.6%)	─			
Non-English	174 (19.4%)	37 (15.4%)	0.75 (0.51–1.10)			
Total travel time (min.), mean (SD)	64.4 (64.5)	68.0 (98.0)	1.02 (0.96–1.08)^c^	0.50		
Total distance (mile), mean (SD)	41.3 (59.9)	47.7 (90.5)	1.00 (1.00–1.01)^d^	0.21		
ADI score (quartile)				0.20		
1 st	337 (37.6%)	79 (32.8%)	─			
2nd	197 (22.0%)	67 (27.8%)	1.45 (1.00–2.10)			
3rd	206 (23.0%)	59 (24.5%)	1.22 (0.83–1.78)			
4th	157 (17.5%)	36 (14.9%)	0.98 (0.63–1.50)			

OR, odds ratio; CI, confidence interval; SD, standard deviation; EOT, end of treatment; CNS, central nervous system; ADI, area deprivation index; RT, radiation therapy

^a^Defined as ≥ 1 LTSC visit at least 2 years from end of treatment, with an ending follow-up date of December 31, 2024

^b^Defined as no LTSC visit at least 2 years from end of treatment, with an ending follow-up date of December 31, 2024

^c^OR for travel time is per 30-min increase

^d^OR for total distance is per 2-mile increase

**Fig. 1:**
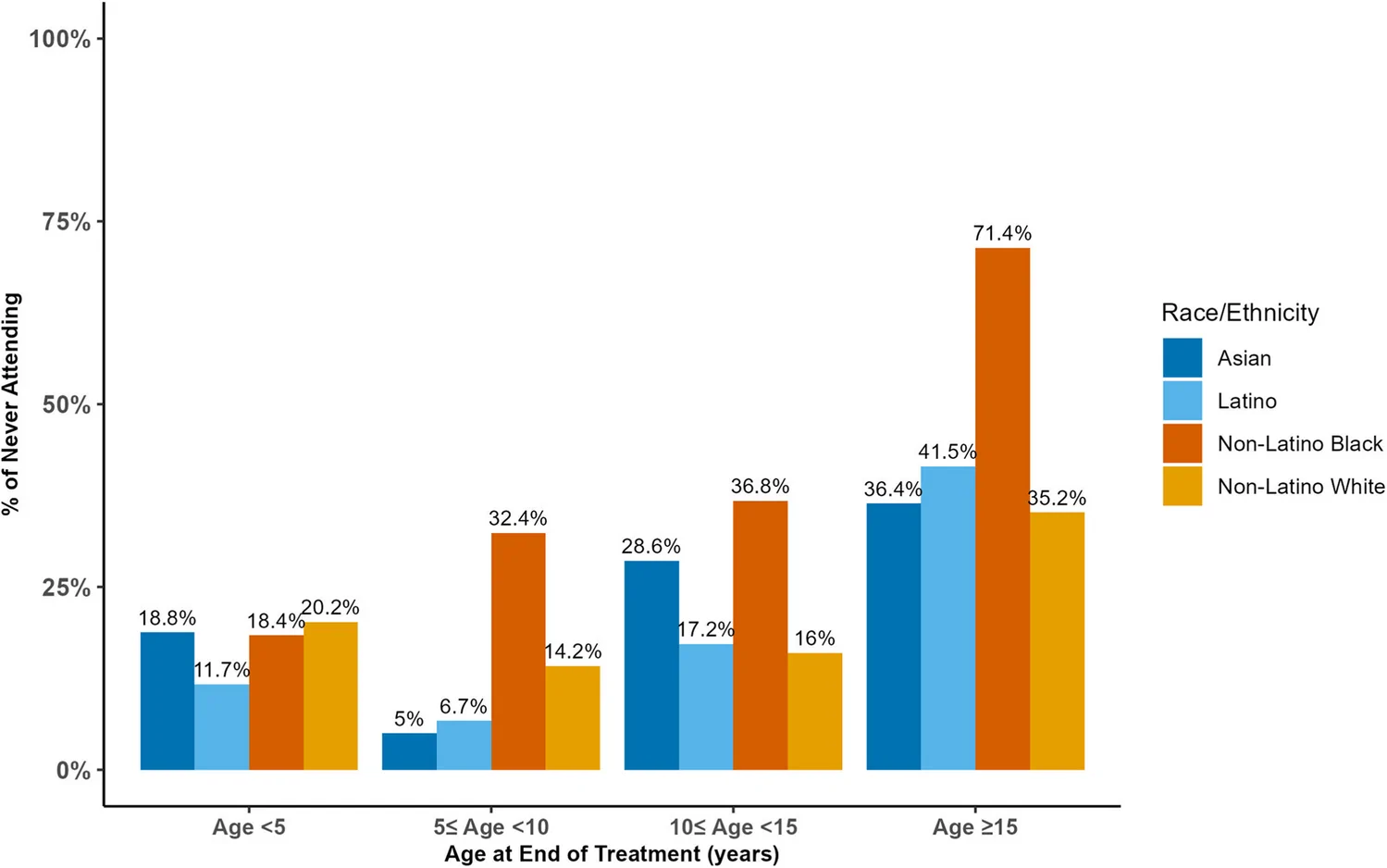
The proportion of survivors who never attended LTSC by age at end of treatment, race, and ethnicity

Among eligible survivors, 21% had never attended LTSC (Table 1). In adjusted analyses, LTSC nonattendance was more frequent among older survivors at the end of treatment (adjusted OR [aOR] = 1.15, 95% CI = 1.11–1.18). LTSC nonattendance was less frequent in Latino survivors compared to non-Latino white survivors (aOR = 0.56, 95% CI = 0.37–0.84), and more frequent among survivors with public insurance or no insurance compared to those with commercial insurance (aOR = 2.37; 95% CI, 1.65–3.44 and aOR = 2.58; 95% CI, 1.20–5.42, respectively). Diagnoses of CNS tumor, lymphoma, or solid tumor were associated with a higher likelihood of LTSC nonattendance compared to leukemia (aOR = 2.73; 95% CI, 1.33–5.43; aOR = 2.49; 95% CI, 1.57–3.95; and aOR = 5.23; 95% CI, 3.44–8.06, respectively). Those treated with both chemotherapy and radiation had a lower likelihood of LTSC nonattendance vs. chemotherapy alone (aOR = 0.60; 95% CI, 0.40–0.90). We identified a borderline significant interaction between age at the end of treatment and race/ethnicity in the association of LTSC nonattendance (*P* for interaction = 0.049). Data shown in Fig. 1 demonstrates that in our population, non-Latino Black survivors experience the largest increase in the prevalence of LTSC nonattendance, particularly in those with older age at the end of treatment. No other significant interactions were observed. We also examined these associations with time to LTS attendance using a Cox proportional hazards model, and the results were consistent with those from the logistic regression model (Supplementary Table 1).

Table 2 presents the characteristics of 897 survivors who attended at least one LTSC visit, stratified by whether the LTSC visit occurred within 5 years of end of treatment. The average time from the end of treatment to the initial visit was 4.0 years (range, 1.8–10.1 years), with considerable variation by cancer type: mean of 3.1 years for leukemia, 3.9 years for lymphoma, 5.0 years for solid tumor, and 5.6 years for CNS tumors. Twenty-six percent of survivors who attended LTSC at least once did so with a delayed initiation (first visit beyond 5 years after end of treatment). In the multivariable analysis, older age at end of treatment was associated with lower odds of delayed LTSC visit initiation (aOR = 0.95; 95% CI, 0.92–0.99), as was having a primary language other than English. In contrast, survivors diagnosed with a CNS tumor, lymphoma, or solid tumor had higher odds of delayed LTSC visit initiation compared with leukemia (aOR = 32.70, 95% CI 17.90–62.10; aOR = 5.86, 95% CI 3.11–11.20; and aOR = 19.80, 95% CI 11.90–34.60, respectively).

**Table 2:** Univariable and multivariable analyses of factors associated with delayed long-term survivor clinic (LTSC) visit initiation following cancer treatment (*n* = 897)

Variable	Early LTSC Visit Initiation^a^ (*n* = 665)	Delayed LTSC Visit Initiation^b^ (*n* = 232)	Crude OR(95% CI)	*P*–value	Adjusted OR (95% CI)	*P*–value
Age at end of treatment (years), mean (SD)	9.3 (5.2)	6.8 (5.0)	0.91 (0.88–0.94)	< 0.001	0.95 (0.92–0.99)	0.02
Sex				0.11		0.07
Male	367 (55.2%)	142 (61.2%)	─		─	
Female	298 (44.8%)	90 (38.8%)	0.78 (0.57–1.06)		0.71 (0.49–1.02)	
Race/ethnicity^c^				0.03		0.08
Non-Latino White	203 (30.5%)	90 (38.8%)	─		─	
Asian	37 (5.6%)	7 (3.0%)	0.43 (0.17–0.94)		0.48 (0.17–1.23)	
Latino	356 (53.5%)	113 (48.7%)	0.72 (0.52–0.99)		1.22 (0.80–1.87)	
Non-Latino Black	62 (9.3%)	22 (9.5%)	0.80 (0.46–1.37)		0.64 (0.33–1.20)	
Language				0.03		0.04
English	525 (78.9%)	198 (85.3%)	─		─	
Non-English	140 (21.1%)	34 (14.7%)	0.64 (0.42–0.96)		0.58 (0.33–0.98)	
Diagnosis				< 0.001		< 0.001
Leukemia	403 (60.6%)	20 (8.6%)	─		─	
CNS tumor	39 (5.9%)	61 (26.3%)	31.50 (17.60–58.80)		32.70 (17.90–62.10)	
Lymphoma	107 (16.1%)	27 (11.6%)	5.08 (2.76–9.52)		5.86 (3.11–11.20)	
Solid tumors	116 (17.4%)	124 (53.4%)	21.50 (13.10–37.00)		19.80 (11.90–34.60)	
Payer status 2–6 years from EOT				0.90		
Commercial	319 (48.0%)	114 (49.1%)	─			
Public	325 (48.9%)	110 (47.4%)	0.95 (0.70–1.28)			
Uninsured	21 (3.2%)	8 (3.4%)	1.07 (0.43–2.39)			
Treatment modality				< 0.001		
Chemotherapy only	527 (79.2%)	127 (54.7%)	─			
Chemotherapy + RT	130 (19.5%)	84 (36.2%)	2.68 (1.91–3.75)			
RT only	8 (1.2%)	21 (9.1%)	10.90 (4.89–26.70)			
Total travel time (min.), mean (SD)	62.7 (58.8)	69.2 (78.6)	1.04 (0.98–1.11)^d^	0.20		
Total distance (miles), mean (SD)	37.0 (48.0)	53.7 (84.3)	1.01 (1.00–1.01)^e^	0.001		
ADI score (quartile)				0.40		
1 st	259 (38.9%)	78 (33.6%)	─			
2nd	143 (21.5%)	54 (23.3%)	1.25 (0.84–1.87)			
3rd	146 (22.0%)	60 (25.9%)	1.36 (0.92–2.02)			
4th	117 (17.6%)	40 (17.2%)	1.14 (0.73–1.75)			

*OR* odds ratio, *CI* confidence interval, *SD* standard deviation, *EOT* end of treatment, *CNS* central nervous system, *ADI* area deprivation index, *RT* radiation therapy

^a^Defined as LTSC attendance within 2–5 years from end of treatment

^b^Defined as LTSC attendance more than 5 years from end of treatment

^c^“Other” category has been removed due to the absence of observations in “delayed LTSC visit initiation” category

^d^OR for travel time is per 30-min increase

^e^OR for total distance is per 2-mile increase

Among survivors who attended LTSC, those with delayed LTSC visit initiation were more likely to only be seen once, vs. survivors with repeated LTSC visits (Table 3, 34% vs. 22%). However, this association did not retain significance in the multivariable model. LTSC visit disengagement with survivorship care (survivors seen once and never again) was more frequent among survivors who were older at the end of treatment (aOR = 1.09; 95% CI, 1.05–1.12) and among those with public insurance (aOR = 2.29; 95% CI, 1.55–3.42). Additionally, survivors diagnosed with CNS tumor, lymphoma, or solid tumor had a higher odd of LTSC visit disengagement compared to those diagnosed with leukemia (aOR = 3.26, 95% CI, 1.84–5.76; aOR = 2.90, 95% CI, 1.81–4.65; and aOR = 3.27, 95% CI, 2.06–5.22, respectively).

**Table 3:** Univariable and multivariable analyses of factors associated with long-term survivor clinic (LTSC) visit disengagement following cancer treatment (*n* = 875)

Variable	LTSC visit engagement^a^ (*n* = 659)	LTSC visit disengagement^b^ (*n* = 216)	Crude OR(95% CI)	*P*–value	Adjusted OR (95% CI)	*P*–value
Delayed LTSC Visit Initiation	146 (22.2%)	73 (33.8%)	1.79 (1.28–2.51)	< 0.001	1.28 (0.85–1.92)	0.24
Age at end of treatment (years), mean (SD)	8.3 (5.0)	9.7 (5.8)	1.05 (1.02–1.08)	0.001	1.09 (1.05–1.12)	< 0.001
Sex				0.3		0.45
Male	367 (55.7%)	129 (59.7%)	─		─	
Female	292 (44.3%)	87 (40.3%)	0.85 (0.62–1.16)		0.88 (0.63–1.23)	
Race/ethnicity				0.12		0.22
Non-Latino White	213 (32.3%)	71 (32.9%)	─		─	
Asian	35 (5.3%)	9 (4.2%)	0.77 (0.33–1.62)		0.91 (0.38–2.01)	
Latino	354 (53.7%)	104 (48.1%)	0.88 (0.62–1.25)		0.72 (0.47–1.10)	
Non-Latino Black	52 (7.9%)	30 (13.9%)	1.73 (1.02–2.91)		1.37 (0.77–2.42)	
Other	5 (0.8%)	2 (0.9%)	1.20 (0.17–5.70)		0.74 (0.1.–3.77)	
Language				0.30		0.50
English	526 (79.8%)	179 (82.9%)				
Non-English	133 (20.2%)	37 (17.1%)	0.82 (0.54–1.21)		0.85 (0.52–1.36)	
Payer status 2–6 years from EOT				0.02		< 0.001
Commercial	336 (51.0%)	86 (39.8%)	─		─	
Public	303 (46.0%)	121 (56.0%)	1.56 (1.14–2.15)		2.29 (1.55–3.42)	
Uninsured	20 (3.0%)	9 (4.2%)	1.76 (0.74–3.89)		1.97 (0.76–4.83)	
Diagnosis				< 0.001		< 0.001
Leukemia	356 (54.0%)	59 (27.3%)	─		─	
CNS tumor	60 (9.1%)	35 (16.2%)	3.52 (2.13–5.79)		3.26 (1.84–5.76)	
Lymphoma	83 (12.6%)	50 (23.1%)	3.63 (2.33–5.68)		2.90 (1.81–4.65)	
Solid tumors	160 (24.3%)	72 (33.3%)	2.72 (1.84–4.03)		3.27 (2.06–5.22)	
Treatment modality				0.002		
Chemotherapy only	502 (76.2%)	140 (64.8%)	─			
Chemotherapy + RT	142 (21.5%)	64 (29.6%)	1.62 (1.14–2.29)			
RT only	15 (2.3%)	12 (5.6%)	2.87 (1.29–6.26)			
Total travel time (min.), mean (SD)	62.8 (62.7)	69.5 (69.4)	1.05 (0.98–1.12)^c^	0.20		
Total distance (mile), mean (SD)	40.8 (61.1)	42.8 (56.7)	1.00 (1.00–1.01)^d^	0.68		
ADI score (quartile)				0.04		
1 st	264 (40.1%)	65 (30.1%)	─			
2nd	136 (20.6%)	58 (26.9%)	1.73 (1.15–2.61)			
3rd	143 (21.7%)	55 (25.5%)	1.56 (1.03–2.36)			
4th	116 (17.6%)	38 (17.6%)	1.33 (0.84–2.09)			

*OR* odds ratio, *CI* confidence interval, *SD* standard deviation, *EOT* end of treatment, *CNS* central nervous system, *ADI* area deprivation index, *RT* radiation therapy

^a^Defined as LTSC attendance more than once

^b^Defined as LTSC attendance only once

^c^OR for travel time is per 30-min increase

^d^OR for total distance is per 2-mile increase

## Discussion

Within this demographically and socioeconomically diverse patient population, we characterize factors associated with LTSC nonattendance, delayed LTSC visit initiation, and LTSC visit disengagement among survivors of cancer diagnosed in childhood and adolescence. Seventy-nine percent of survivorship-eligible survivors diagnosed 2011–2019 initiated LTSC at TXCH. This attendance exceeds rates reported by other similar childhood cancer survivorship programs in the United States that, similar to TXCH, lack a philanthropy-based payer system[5, 6, 8, 20–24] and approaches the 85% attendance rate reported by the After Completion of Therapy Clinic at St. Jude Children’s Research Hospital[25], which provides substantive financial resources to patients to support their travel to participate in survivor-focused care. Many of our findings were similar to published studies indicating higher likelihood of LTSC attendance among Latino survivors[6], those treated with both chemotherapy and radiation (vs. surgery alone)[5, 6, 21, 26], and those who are privately insured[22, 24]. In our population, patients treated with radiation alone were less likely to attend LTSC, which may reflect fragmentation in care as radiation is provided to TXCH patients through a partner institution. Our findings were also concordant with published reports suggesting lower LTSC attendance among older survivors[6, 8, 20–24], non-White[20, 25] or Black survivors[5, 21], survivors with a non-leukemia diagnoses[8, 23], and survivors who are uninsured[6, 21–23, 25] or who have public insurance[5, 22, 23]. In contrast with published reports, our study did not find an association between LTSC attendance and distance traveled[5, 8, 21, 23, 26, 27] or with area-based deprivation[24, 26] and other indicators of socioeconomic status[27].

Our results also provide novel insight into follow-up patterns among survivors treated for cancer in childhood, demonstrating the negative impact of delayed initiation of survivorship care on continued survivor engagement with the clinic. In our study population, survivors who initiated survivorship care more than 5 years after the end of cancer treatment were more likely not to return for continued survivorship care. Moreover, survivors of childhood leukemia were among those most likely to initiate care early after treatment completion, and thus also most likely to remain engaged. Conversely, survivors of childhood solid tumors or CNS tumors were less likely to attend LTSC, initiate care early, or maintain engagement, which is consistent with findings from previous studies[7, 8]. Differences in survivorship care by cancer type may be influenced by system-level factors and institutional practices[7]. For example, survivors of CNS tumors may be directed to subspecialty clinics rather than survivorship clinics[8, 28]. Local institutional practice may also contribute to the superior attendance and engagement observed among leukemia survivors, i.e., a standard of practice adopted in 2018 that outlines a 5-year-long shared care model between the primary oncologist and the LTSC provider for the transition of patients with acute lymphoblastic leukemia (ALL) and acute myeloid leukemia (AML) from treatment to survivorship. Once children with ALL and AML complete treatment, they are moved to an institutional practice standard that specifically addresses the transitional time from leukemia treatment to survivorship. The treatment plan includes off therapy evaluations (such as complete blood counts and liver function tests) as well as a reminder to refer to the LTSC at 24 months from end of therapy. This approach is unique to leukemia survivors at our center and exemplifies characteristics marking successful care transitions in pediatric populations, e.g., having an established diagnosis and/or medication needs, initiating transition when patients are still requiring more frequent than annual follow-up, and introducing transition planning early[29–31]. Given the apparent benefits of this primary oncology/LTSC shared care approach to improving LTSC attendance and engagement, we plan to expand this practice to include other TXCH cancer populations, including solid tumors and CNS tumors, and recommend that pediatric oncology clinics consider adopting a similar model in transitioning patients from treatment to survivorship.

Several factors contributing to nonattendance in our population are well-known, such as older age at diagnosis or end of treatment. Adolescents and young adults are a highly mobile population and particularly vulnerable to the financial, social and psychological impacts of the cancer experience[32]. We also note that public insurance and uninsured status were associated with LTSC nonattendance and LTSC disengagement, recapitulating previous findings of lower LTSC attendance and engagement among older, publicly insured survivors[24]. Insurance-related disparities with respect to access to health services are most prominent in non-Medicaid expansion states such as Texas[33]. Moreover, while the Affordable Care Act offers protections to individuals aged 18 to 25 years old and to those with chronic health conditions[34], many survivors experience insurance instability and disruptions as they enter adulthood[33], which is known to impact survivor access to preventive care and treatment as well as their survival after cancer[35]. We also observed Latino survivors as most likely to attend LTSC. A prior study conducted in Latino survivors of childhood cancer identified two factors that were strongly predictive of intent to receive follow-up care in the next 2 years were greater self-efficacy and having health insurance[36]. One possible explanation for this effect in our study population is the prevalence of Spanish-speaking providers and staff as well as in-person language services that are available to support Latino access to care. Early entry to survivorship care, e.g., 2–5 years from end of treatment, provides a critical opportunity to promote self-efficacy as well as to identify and address anticipated changes in residence, insurance status or payer, vocational or educational status and financial wellness as survivors enter adulthood. As observed by other groups, non-Latino Black survivors, particularly older survivors, were least likely to be seen in LTSC. Qualitative assessment of factors driving this observation, including an evaluation of insurance related barriers, is needed to inform tailored approaches to engagement and education to address this disparity.

Study strengths include the large sample size, diverse population, lifelong access to survivorship care that promotes inclusion of cancer survivors across the lifespan, evaluation of a broad spectrum of factors contributing to LTSC attendance, e.g., language spoken, distance traveled, and area-based measures, and inclusion of factors predictive of survivor engagement beyond the first LTSC visit. This study also has some limitations. The study time frame was designed to begin with the year of EPIC® implementation at Texas Children’s Hospital to facilitate data abstraction: decades of LTSC data from years prior to 2011 were not available for analysis. Many of the patients diagnosed from 2011 to 2019 were seen in LTSC during the COVID-19 pandemic, during which time telehealth visits were more prevalent at our center and elsewhere[37]. Though we did not distinguish telehealth from in-person visits for this study, there are advantages to telehealth as a more accessible means for obtaining survivorship services[38], and this may have impacted attendance rates. We did not include survivors treated with surgery alone, nor did we include those who are treated with allogeneic transplantation and are at an exceptionally high risk for late effects, early morbidity, and mortality. Although this is a single institution study, TXCH is a referral center for childhood cancer, with 500–600 children diagnosed each year in a diverse population that is ~ 50% publicly insured and ~ 11% rural. Our internal referral practices and population may limit the generalizability of the findings to the broader pediatric cancer survivor population. Data utilized to determine payer status and area-based measures were collected from the electronic health record, which necessitates contact with the hospital system and may not reflect interim gaps in insurance coverage or changes in residence that occur between contact points with Texas Children’s. Limiting our analysis to the first and most recent LTSC visits precludes a more detailed understanding of adherence to annual follow-up, which would require a more in-depth investigation that includes longitudinal payer data, residence, and area-based assessment over time.

## Conclusion

The results of this study add to a growing body of evidence that at least one in five survivors are not transitioning to survivor-focused health care after treatment. Our findings suggest that a key intervention supporting both LTSC attendance and ongoing engagement is the early, intentional transition from treatment to survivorship[39], i.e., initiation of survivorship care during the disease surveillance period and alternating visits between the treatment and survivorship clinical teams. We acknowledge that this approach may not be feasible for pediatric oncology clinics that lack a designated survivorship clinician or clinic service or that have an upper age boundary of service eligibility, and in those cases encourage early discussions of the survivorship care plan once cancer treatment is completed and before referral to the community for long-term follow-up care. Future studies should consider investigating barriers related to the transition from pediatric to adult-based survivorship care and related determinants to understand challenges uniquely associated with lifelong models of care. Access to survivor-specific education and medical care is a critical unmet need in the childhood cancer survivor community[3]. By identifying factors associated with failure to initiate and maintain survivorship care, we can design more effective interventions that address barriers and improve care delivery and health outcomes.

## Supplementary Information

Below is the link to the electronic supplementary material.

ESM1 (DOCX 25.3 KB)

## Data Availability

Data is available upon request due to privacy/ethical restrictions
