# Supplementary material for: Predictors of long-term follow-up clinic attendance and continued engagement among survivors of cancer in childhood and adolescence
Source: J Cancer Surviv. 2025 Oct 15;20(5):2085–94. doi: 10.1007/s11764-025-01903-4 (PMC12696749; doi:10.1007/s11764-025-01903-4)
Supplement: Supplementary file 1 [file 11764_2025_1903_MOESM1_ESM.docx]

Supplementary Figure 1. Flowchart of study population

Study Population: Childhood cancer survivors diagnosed 2011-2019 and treated at TXCH (n=1194)

Inclusion Criteria:

1) Treated with radiation therapy, chemotherapy, or both

2) Alive and in remission >2 years after EOT

3) Reside within a 300-mile radius of TXCH

Exclusion Criteria:

1) Refractory disease or relapse

2) Transferred care to another hospital within 2 years from EOT

3) Treated with observation or surgery alone

4) Treated with an allogeneic hematopoietic stem cell transplantation

4) International referrals

5) No insurance data at 2-6 years from EOT or international payer status

Survivors included in the study (n=1138)

Analysis for Outcome 1: LTSC non-attendance

Analysis for Outcome 2: LTSC visit delayed initiation (n= 897)

Analysis for Outcome 3: LTSC visit disengagement (n=875)

Exclusion Criteria:

Patients who never attended (n=241)

Exclusion Criteria:

Patients who attended their first LTSC visit but then relapsed, died, or moved away within 5 years of EOT (n=22)

| **Supplementary Table 1**. Time-to-Event Analyses of Factors Associated with Long-Term Survivor Clinic (LTSC) Nonattendance Following Cancer Treatment (n=1138) | |
| --- | --- |
| Variable | HR  (95% CI) |
| Age at end of treatment (continuous) | 1.05 (1.03-1.06) |
| Sex |  |
| Male | ─ |
| Female | 1.02 (0.89-1.16) |
| Race/ethnicity |  |
| Non-Latino White | ─ |
| Asian | 0.88 (0.64-1.21) |
| Latino | 0.79 (0.67-0.93) |
| Non-Latino Black | 1.23 (0.96-1.58) |
| Other | 0.96 (0.45-2.04) |
| Payer status 2-6 years from EOT |  |
| Commercial | ─ |
| Public | 1.33 (1.15-2.04) |
| Uninsured | 1.67 (1.14-2.44) |
| Treatment modality |  |
| Chemotherapy only | ─ |
| Chemotherapy and radiation | 0.83 (0.69-0.99) |
| Radiation only | 1.52 (0.98-2.37) |
| Diagnosis |  |
| Leukemia | ─ |
| CNS tumor | 3.03 (2.30-4.00) |
| Lymphoma | 2.11 (1.72-2.60) |
| Solid tumors | 3.51 (2.95-4.19) |
| HR, hazard ratio; CI, confidence interval; EOT, end of treatment; CNS, central nervous system. | |
